# Alteration in Gastric Ghrelin Expression in a Mouse Model of Type 2 Diabetes Mellitus

**DOI:** 10.7759/cureus.96035

**Published:** 2025-11-03

**Authors:** Minami Watanabe, Ippei Watari, Masato Akakura, Srisutha Jiratchaya, Takashi Ono

**Affiliations:** 1 Department of Orthodontic Science, Institute of Science Tokyo, Tokyo, JPN; 2 Department of Orthodontics, Chulalongkorn University, Bangkok, THA

**Keywords:** ghrelin, insulin, mouse model, stomach, type 2 diabetes mellitus

## Abstract

Background

Ghrelin, produced and secreted in the stomach, stimulates appetite by acting on the hypothalamus and inhibits insulin secretion, leading to hyperglycemia. Ghrelin is also associated with obesity and insulin resistance in type 2 diabetes mellitus (T2DM). Beyond metabolic regulation, ghrelin is linked to autophagy and oxidative stress, leading to many chronic inflammatory diseases. However, few studies have focused on the local mechanisms of ghrelin secretion in a diabetic stomach. We aimed to investigate ghrelin expression in the gastric mucosa using a mouse model of T2DM.

Methods

At five weeks of age, male C57BL/6J mice (Japan SLC, Hamamatsu, Japan) were randomly assigned to two major groups: a T2DM (DM) group and an age-matched control (CON) group. The DM group was fed a high-fat diet from six weeks of age, and the CON group was fed a normal diet. At week four, the DM mice received intraperitoneal injections of streptozotocin (STZ). Gastric mucosa samples were collected at 11 weeks and 13 weeks of age, and ghrelin mRNA expression was analyzed using reverse transcription-quantitative polymerase chain reaction (RT-qPCR). Only samples that met the quality criteria (A260/A280 = 1.8-2.0 and RNA integrity number (RIN) > 7) were included in the RT-qPCR analysis, resulting in n = 4 (11 weeks) and n = 5 (13 weeks) per group. The sample size was determined based on feasibility and reference to similar studies. All experimental procedures were approved by the Institutional Animal Care and Welfare Committee of Tokyo Medical and Dental University, Tokyo, Japan (approval no.: A2022-090A).

Results

The DM group showed significantly higher body weight and blood glucose levels than the CON group. Ghrelin mRNA expression significantly increased in the 13-week-old DM group compared to the CON group. No significant difference was observed at 11 weeks.

Conclusions

Immunostaining and RT-qPCR analyses revealed elevated gastric ghrelin levels in T2DM mice. This first demonstration of ghrelin upregulation in the diabetic gastric mucosa emphasizes the importance of assessing local gastric expression, which has been largely overlooked compared with circulating levels. These findings suggest a compensatory role of gastric ghrelin in endocrine regulation and may provide translational insights that could contribute to the development of novel therapeutic approaches for diabetes.

## Introduction

Type 2 diabetes mellitus (T2DM) is a chronic disease caused by obesity and lifestyle changes [1, 2]. The primary mechanisms involve decreased insulin secretion and resistance, and prolonged hyperglycemia induces oxidative stress that damages cells and leads to apoptosis of enteric neurons. The resulting neuronal loss reduces colonic motility, causing gastrointestinal symptoms such as constipation and diarrhea, including diabetic gastroparesis, characterized by delayed gastric emptying. These gastrointestinal disturbances impair digestion and worsen glycemic control, thereby reducing the quality of life for patients with T2DM [3, 4]. Ghrelin was first identified in 1999 as an endogenous ligand for the growth hormone secretagogue receptor in rat stomachs [5]. Plasma ghrelin levels decrease in obesity, contributing to insulin resistance [6]. While numerous studies have examined the systemic metabolic roles of ghrelin, including appetite regulation, lipid metabolism, and glucose homeostasis, these aspects provide limited insight into its local gastric regulation. In addition, ghrelin is associated with autophagy, oxidative stress, and inflammation, suggesting a link to gastrointestinal and endocrine alterations in diabetes [7-9].

Ghrelin activates hypothalamic neural circuits to stimulate appetite, promote fat accumulation, and increase body weight. Ghrelin receptor-knockout mice show a loss of appetite-stimulating effects and increased energy expenditure [8]. Moreover, ghrelin stimulates other hormonal pathways, activates anti-inflammatory factors, and inhibits the production of inflammatory factors, contributing to tissue protection [9]. Recently, mastication has gained attention for its role in restoring gastric function. Chewing enhances intestinal motility by stimulating the vagus nerve, increasing myoelectric activity, and promoting gastric acid and pepsinogen secretion [10, 11]. As ghrelin is secreted mainly by the stomach, it likely mediates these vagally driven changes, linking mastication with endocrine and digestive regulation. Decreased circulating ghrelin levels influence the growth hormone (GH)/insulin-like growth factor-1 (IGF-1) axis, potentially leading to increased insulin resistance and the development of T2DM. However, studies have reported both reduced and elevated plasma ghrelin levels in diabetes, depending on the model and disease stage [12, 13]. Such inconsistencies highlight the need to focus on gastric-local expression to clarify ghrelin’s tissue-specific regulation.

The stomach is the primary site of ghrelin production. However, few studies have focused on the local mechanisms of ghrelin secretion in a T2DM stomach. Given that the stomach accounts for most of the ghrelin synthesis, elucidating its local regulation under diabetic conditions remains essential for understanding endocrine adaptations in metabolic disease. Clarifying gastric ghrelin dynamics at the tissue level may provide direct and consistent insights into the role of ghrelin in the pathophysiology of T2DM. Based on these considerations, we hypothesized that ghrelin expression at both the mRNA and protein levels would be altered in the gastric mucosa of streptozotocin (STZ)-induced T2DM mice, with an expected increase in immunohistochemical expression reflecting a compensatory endocrine response to oxidative stress. To test this hypothesis, we aimed to investigate the histological and biochemical aspects of gastric ghrelin secretion in STZ-induced T2DM mice. A previous report on purinergic receptor expression in the submandibular gland of a T2DM mouse model [14] validated this experimental approach, which we adopted for the present study. Nevertheless, no studies have clarified how ghrelin expression is altered in gastric tissue. This study followed the Animal Research: Reporting of In Vivo Experiments (ARRIVE) 2.0 guidelines (Items 1-10b) for transparent animal research reporting (Appendix A) [15].

## Materials and methods

Animals and experimental design

All experimental procedures involving animals were approved by the Institutional Animal Care and Welfare Committee of Tokyo Medical and Dental University, Tokyo, Japan (approval number: A2022-090A). We used 24 male C57BL/6J mice, which were acquired from Sankyo Labo Service (Tokyo, Japan) at five weeks of age. The mice were randomly assigned to the control (CON) group, which received a standard diet (CE-2; CLEA, Tokyo, Japan), and the diabetic (DM) group, which received a high-fat diet (HFD-32; CLEA, Tokyo, Japan). Each cage contained three mice. Both groups were housed under identical environmental conditions (temperature, humidity, etc.). Stomach tissues were fixed in 4% paraformaldehyde (Mildform®; Wako Pure Chemical Industries, Osaka, Japan) for 24 h. The samples were then dehydrated through a graded alcohol series and embedded in paraffin using an RH-12DM embedding machine (Sakura Finetek, Tokyo, Japan) following standard protocols. Embedded tissues were sectioned at 5 μm thickness for mounting on glass slides and then stained with H&E and processed for immunohistochemistry (IHC) and light cycle, differing only in diet composition, to ensure comparability between control and diabetic conditions.

The study design was based on previous reports using STZ-induced diabetic mouse and rat models, where each experimental group typically included six animals for hormonal and histological analyses [13, 16]. Accordingly, our experiment was initially designed with six animals per group. However, one mouse from each group was excluded due to poor general condition unrelated to the experimental treatment, resulting in five animals per group for the final analysis. An a priori power analysis using G*Power 3.1 (Heinrich-Heine-Universität Düsseldorf, Düsseldorf, Germany; two-tailed t-test, α = 0.05, power = 0.80) indicated that approximately eight animals per group would be required to detect an expected large effect size (Cohen’s d ≈ 1.5). Therefore, the final sample size was considered acceptable for detecting major group differences while maintaining ethical reduction principles. Randomization was performed using Microsoft Excel-generated random numbers (Microsoft Corp., Redmond, WA), and all investigators conducting histological and molecular analyses were blinded to the group allocation to minimize bias.

This study followed the ARRIVE 2.0 guidelines (Items 1-10b) for transparent animal research reporting [15].

Following acclimation, the mice were allocated into either a control group receiving a standard diet (CE-2; CLEA, Tokyo, Japan) or a diabetes model group receiving a high-fat diet (HFD-32; CLEA). Each group was further subdivided for euthanasia at either 11 or 13 weeks of age. A total of 24 male C57BL/6J mice were initially used (n = 6 per group). Due to exclusion based on health status and RNA quality control (RNA integrity number (RIN) > 7), the final numbers included in all analyses were n = 4 per group at 11 weeks and n = 5 per group at 13 weeks.

The T2DM model was induced using a high-fat diet combined with a low-dose STZ injection, following the established protocol by Srisutha et al. [14]. In brief, after four weeks on their respective diets, the DM groups received a single intraperitoneal injection of STZ (40 mg/kg in 0.05 M citrate buffer, pH 4.5), while control groups received the citrate buffer.

All mice were fasted for four hours prior to injection, in accordance with the National Institutes of Health (NIH) Diabetes Complications Consortium protocol (2025) [17]. Body weight was measured every two weeks throughout the study.

**Figure 1:**
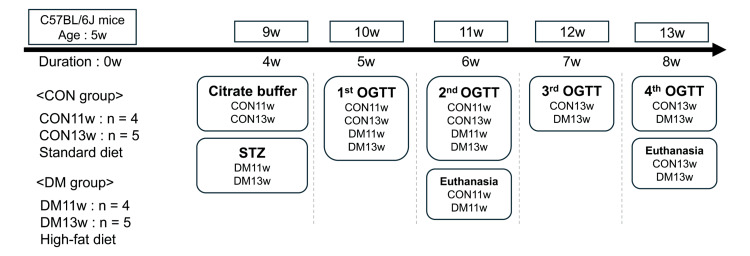
Experimental design At five weeks of age, male C57BL/6J mice were randomly assigned to four experimental groups: CON11w, CON13w, DM11w, and DM13w. CON groups were fed a standard diet (SD), and DM groups received a high-fat diet. At nine weeks of age, the DM11w and DM13w groups were injected with streptozotocin (STZ), and the CON11w and CON13w groups were injected with citrate buffer. Oral glucose tolerance tests (OGTT) were conducted weekly after injection. Mice were euthanized at 11 or 13 weeks of age, depending on the group. CON: control group; DM: type 2 diabetes mellitus group; w: weeks

At weeks 6 and 8 of the study (Figure 1), all mice were euthanized, and their stomachs were immediately collected. Gastric tissues were then prepared for further analyses by reverse transcription-quantitative polymerase chain reaction (RT-qPCR), IHC, and H&E staining. H&E-stained sections were examined to confirm histological changes in the gastric tissue after diabetes induction.

Oral glucose tolerance test (OGTT)

To assess glucose tolerance, an OGTT was performed once weekly after STZ injection until euthanasia. Mice were fasted for 18 hours before each test [18]. Baseline blood glucose was measured at 0 min. Then, a 10% D-(+)-glucose solution (Nacalai Tesque, Kyoto, Japan) was administered orally at 1 g/kg (10 μL/g) [19, 20]. Blood glucose levels were recorded at 0, 30, 60, 90, and 120 minutes using an Accu-Chek Guide glucometer (Roche Diagnostics, Basel, Switzerland). At each time point, 5 μL of blood was collected via tail pricking. The area under the glucose curve (AUCglu) was calculated using GraphPad Prism 9 (GraphPad Software, La Jolla, CA).

Histological preparation of the stomach

Stomach tissues were fixed in 4% paraformaldehyde (Mildform®; Wako Pure Chemical Industries) for 24 hours. The samples were then dehydrated through a graded alcohol series and embedded in paraffin using an RH-12DM embedding machine (Sakura Finetek) following standard protocols. Embedded tissues were sectioned at 5 μm thickness for mounting on glass slides and then stained with H&E and processed for IHC.

Histomorphologic evaluation

For H&E staining, sections were first deparaffinized in xylene and rehydrated through graded ethanol (three minutes each), then rinsed in water (three minutes). Sections were stained with hematoxylin for 12 min followed by eosin for 75 s. Histological features of the stomach were examined at 200× and 400× magnification under a Nikon Microphoto-FXA light microscope (Tokyo, Japan), and images were captured with a Nikon DXM1200 digital camera (Kanagawa, Japan).

Morphometric analysis of the stomach

Three sections per stomach were selected for morphometric analysis. Five random fields per section were imaged at 400× magnification. The thickness of the gastric mucosa in the antrum, body, and glandular base was measured using Fiji ImageJ2 software (version 2.9.0; NIH, Bethesda, MD).

Immunohistochemical staining

Immunohistochemical staining to detect ghrelin expression was carried out using the avidin-biotin peroxidase method, following procedures reported in earlier studies [14, 21]. Paraffin sections were first deparaffinized in xylene and sequentially rehydrated through graded ethanol solutions. For antigen retrieval, the slides were immersed in 0.01 M sodium citrate buffer (pH 6.0) and kept at 60℃ overnight. After retrieval, the sections were rinsed three times in 0.1 M phosphate-buffered saline (PBS, pH 7.4). Permeabilization was achieved by incubating the tissue in PBS containing 0.2% Triton X-100 for 10 minutes at 25℃. Endogenous peroxidase activity was blocked by treating the slides with 3% hydrogen peroxide in methanol for 30 minutes at room temperature.

The samples were then washed twice with distilled water and preincubated with normal goat serum for 30 min at 25℃ to reduce nonspecific binding. Subsequently, the slides were incubated overnight at 4℃ with a rabbit anti-ghrelin primary antibody (Ab209790; Abcam, Cambridge, MA, USA) diluted 1:150 in PBS. After three washes in PBS, the sections were exposed to a biotinylated secondary antibody from the VECTASTAIN Elite ABC-HRP kit (Vector Laboratories, Newark, CA, USA) for 30 min at 25℃. This was followed by additional PBS washes and incubation with the ABC reagent for 30 min at 25℃. The antigen-antibody complexes were visualized using the DAB substrate kit (#ab64238; Abcam) for 40 s. Counterstaining was performed with Mayer’s hematoxylin (Fujifilm Wako Pure Chemical Corp), after which the slides were rinsed in tap water for 15 min.

Finally, the sections were dehydrated through graded ethanol and cleared in xylene before mounting with Mount-Quick (Daido Sangyo, Tokyo, Japan). Observations were made with a Nikon Microphoto-FXA light microscope at 400× magnification, and digital images were captured using a Nikon DXM1200 camera. The image scale was calibrated using a stage micrometer (Nikon) under a 40× objective lens prior to image acquisition, yielding a calibration value of 0.16 μm/pixel to ensure accurate measurement of gastric mucosal structures. For each animal, two sections were prepared, and from each section, 20 random microscopic fields (300 × 300 pixels, 0.05 × 0.05 mm) were selected. The number of ghrelin-positive cells in these fields was quantified with Fiji ImageJ2 software. Negative control slides were processed in parallel without the primary antibody to confirm staining specificity; no immunoreactivity was observed (data not shown). Two independent blinded observers quantified the number of ghrelin-positive cells, and inter-observer reliability was assessed using the intraclass correlation coefficient (ICC > 0.9).

RT-qPCR

Immediately after removal, each stomach was snap-frozen at −80℃ and stored until RNA extraction. Total RNA was isolated using an acid guanidinium thiocyanate-phenol-chloroform reagent (Sepasol RNA I Super G; Nacalai Tesque), according to the manufacturer’s protocol. Tissue homogenization was carried out with a disposable homogenizer (BioMasher II; Nippi Inc., Tokyo, Japan) in 1 mL of Sepasol RNA I Super G, and the lysates were left to stand at room temperature for 5 min. Subsequently, 200 μL of chloroform (Fujifilm Wako Pure Chemical Corp) was added, vigorously shaken, and centrifuged at 12,000 × g for 15 min at 4℃. The aqueous phase was carefully transferred to a new tube and mixed with 500 μL of 2-propanol (Fujifilm Wako). After 10 min of incubation at room temperature, the mixture was centrifuged at 12,000 × g for 10 min at 4℃. The resulting pellet was washed with 1 mL of 75% ethanol (Fujifilm Wako), followed by centrifugation at 12,000 × g for 5 min at 4℃. After brief air-drying, the RNA pellet was dissolved in RNase-free water, and RNA concentration and purity were determined spectrophotometrically at 260 nm and 280 nm using a BECKMAN DU-640 spectrophotometer (Beckman Coulter, Brea, CA, USA).

Only RNA samples that met the quality criteria (A260/A280 = 1.8-2.0 and RNA integrity number > 7.0) were used for downstream analysis. Of the originally collected six samples per group, four (11 weeks) or five (13 weeks) samples per group met these criteria and were included in the RT-qPCR analysis.

RT-qPCR was performed using a commercially available reverse transcription kit with random primers (ReverTra Ace qPCR RT Master Mix; TOYOBO, Osaka, Japan) according to the manufacturer’s instructions. Amplification specificity was verified by melting-curve analysis, showing a single sharp peak for each target gene, and the overall procedures were conducted in accordance with the Minimum Information for Publication of Quantitative Real-Time PCR Experiments (MIQE) guidelines (Appendix B) [22]. Quantitative PCR was then performed using TB Green Premix Ex Taq II (TaKaRa Bio, Otsu, Japan) on a real-time PCR instrument (Applied Biosystems 7500 Real-Time PCR System; Thermo Fisher Scientific, Waltham, MA, USA). Each PCR mixture contained the 2× master mix, 10 μM forward and reverse primers, ROX reference dye II (50×), and RNase-free water (BioDynamics, Tokyo, Japan).

Thermal cycling conditions were as follows: an initial denaturation at 95℃ for 30 seconds, followed by 40 cycles of 95℃ for 5 seconds and 60℃ for 34 seconds. Each sample was analyzed in triplicate to ensure reproducibility.

Relative gene expression was calculated using the 2^(-ΔΔCt) method, with glyceraldehyde-3-phosphate dehydrogenase (GAPDH) as the endogenous reference. Amplification efficiency was assumed to be comparable between the target and reference genes in accordance with MIQE guidelines [22]. The oligonucleotide primers for mouse GAPDH and ghrelin were obtained from Thermo Fisher Scientific, and their sequences are listed in Table 1. Expression levels were expressed as fold changes relative to the control groups.

**Table 1:** Primer sequences GAPDH: glyceraldehyde-3-phosphate dehydrogenase; Ghrl: ghrelin

Gene	Forward Primer Sequence (5'-3')	Reverse Primer Sequence (5'-3')
GAPDH	GCATCTTCTTGTGCAGTGCC	TACGGCCAAATCCGTTCACA
Ghrl	TCTCCCCCAGGTCATCTGTC	CCAGCTCCTCCTCTGTCTCT

Statistical analysis 

All statistical analyses were conducted using GraphPad Prism 9 software. Data are expressed as the mean ± standard error of the mean (SEM) together with the 95% confidence interval (CI). The normality of the datasets (body weight, blood glucose, IHC-positive cell counts, and RT-qPCR expression levels) was assessed using the Shapiro-Wilk test and visually confirmed with Q-Q plots. As all data met assumptions of normality and homogeneity of variance, parametric tests were applied.

Comparisons of body weight and blood glucose levels between the CON11w and DM11w groups, as well as between the CON13w and DM13w groups, were performed with unpaired two-tailed Student’s t-tests. Ghrelin mRNA expression and the number of ghrelin-positive cells were analyzed using two-way analysis of variance (ANOVA), followed by Holm-Sidak’s post hoc test for multiple comparisons. Effect sizes were reported to quantify the magnitude of observed differences: Cohen’s d for t-tests and η² for ANOVA. For example, ghrelin mRNA expression showed a large difference between the DM13w and CON13w groups (Cohen’s d = 1.69, 95% CI (0.10-3.28)), while IHC quantification revealed extremely large effects (Cohen’s d = 8.10 at 11w, 5.75 at 13w). Statistical significance was set at p < 0.05. This approach ensures transparency and reproducibility in accordance with the ARRIVE 2.0 guidelines [15].

## Results

Changes in body weight

At baseline (five weeks of age), no significant difference in body weight was observed between the control and diabetic groups (CON11w vs. DM11w: 19.4 ± 0.3 vs. 19.4 ± 0.4 g; p = 0.98).

From six to nine weeks, body weight increased steadily in both groups; however, diabetic mice showed a more pronounced increase, with a significant difference emerging at 9 weeks (CON11w: 25.6 ± 0.5 g vs. DM11w: 28.5 ± 0.6 g; p = 0.012, Cohen’s d = 2.05).

At 10 weeks (immediately before euthanasia), mean body weight remained higher in the diabetic group (CON11w: 22.8 ± 0.4 g vs. DM11w: 25.6 ± 0.5 g; p = 0.018, d = 1.95).

**Figure 2:**
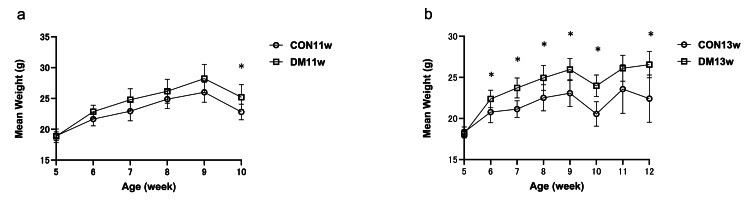
Changes in body weight a: Weekly mean body weight from the CON11w and DM11w groups (n = 4 each); b: values for the CON13w and DM13w groups (n = 5 each). Data are shown as mean ± SEM. An unpaired two-tailed Student’s t-test was used for comparison. * p < 0.05. CON: control group; DM: type 2 diabetes mellitus group; w: weeks

A similar tendency was observed in the 13-week cohort, with the DM13w mice exhibiting persistently greater body weight than their controls throughout the experiment (Figure 2). These results confirm successful induction of type 2 diabetes, accompanied by progressive body weight gain in the high-fat diet groups.

OGTT

OGTT was performed in all groups to assess glucose tolerance. Data are shown as mean ± SEM (n = 4 for 11 w; n = 5 for 13 w). At 11 weeks, blood glucose levels in the DM11w group were significantly higher than those in the CON11w group at 30 min (mean ± SEM: 256.0 ± 13.8 vs. 185.8 ± 12.4 mg/dL; p = 0.016, Cohen’s d = 2.09) and at 120 min (166.8 ± 6.8 vs. 126.5 ± 6.4 mg/dL; p = 0.033, d = 1.74).

At 13 weeks, the DM13w group showed markedly elevated glucose levels compared with the CON13w group at 30 min (311.6 ± 8.2 vs. 215.6 ± 10.4 mg/dL; p = 0.0003, d = 3.74), 60 min (273.8 ± 9.4 vs. 200.4 ± 8.9 mg/dL; p = 0.0044, d = 2.50), and 90 min (245.8 ± 8.2 vs. 194.2 ± 7.9 mg/dL; p = 0.021, d = 1.79).

**Figure 3:**
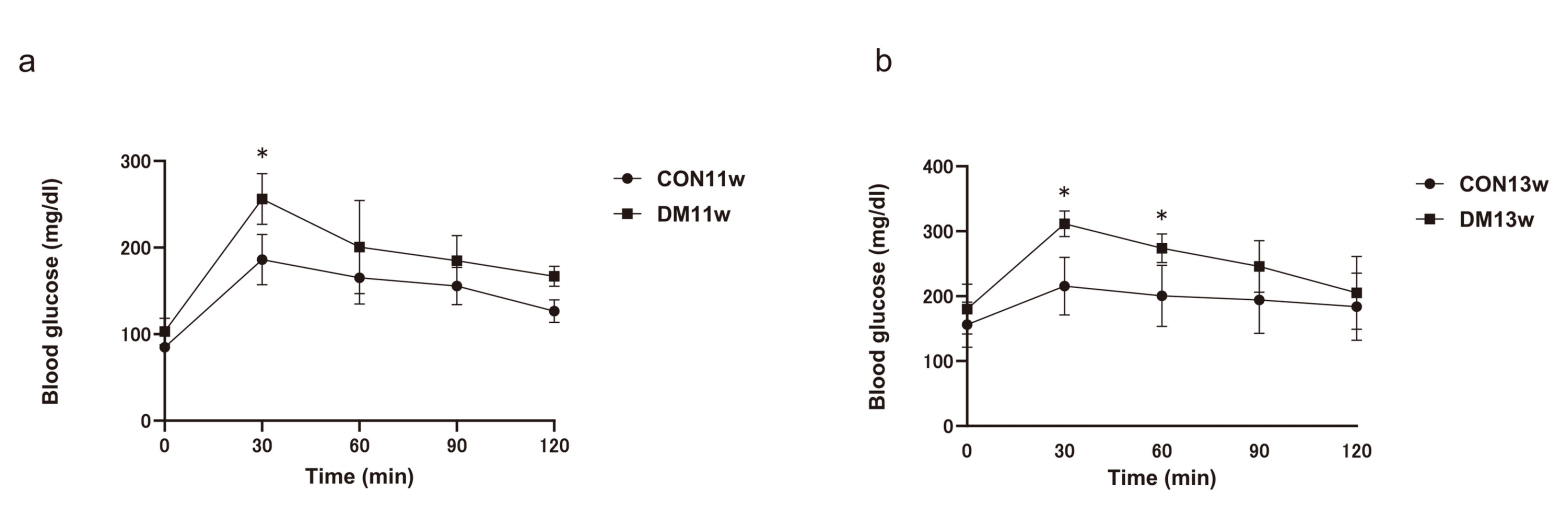
Comparison of the oral glucose tolerance test (OGTT) a: Comparison between the CON11w and DM11w groups (n = 4 each); b: comparison between the CON13w and DM13w groups (n = 5 each). Data are shown as mean ± SEM. An unpaired two-tailed Student’s t-test was used at each time point. * p < 0.05. CON: control group; DM: type 2 diabetes mellitus group; w: weeks

These findings indicate impaired glucose tolerance in the diabetic mice, characterized by a delayed return of glucose levels toward baseline (Figure 3).

H&E staining

**Figure 4:**
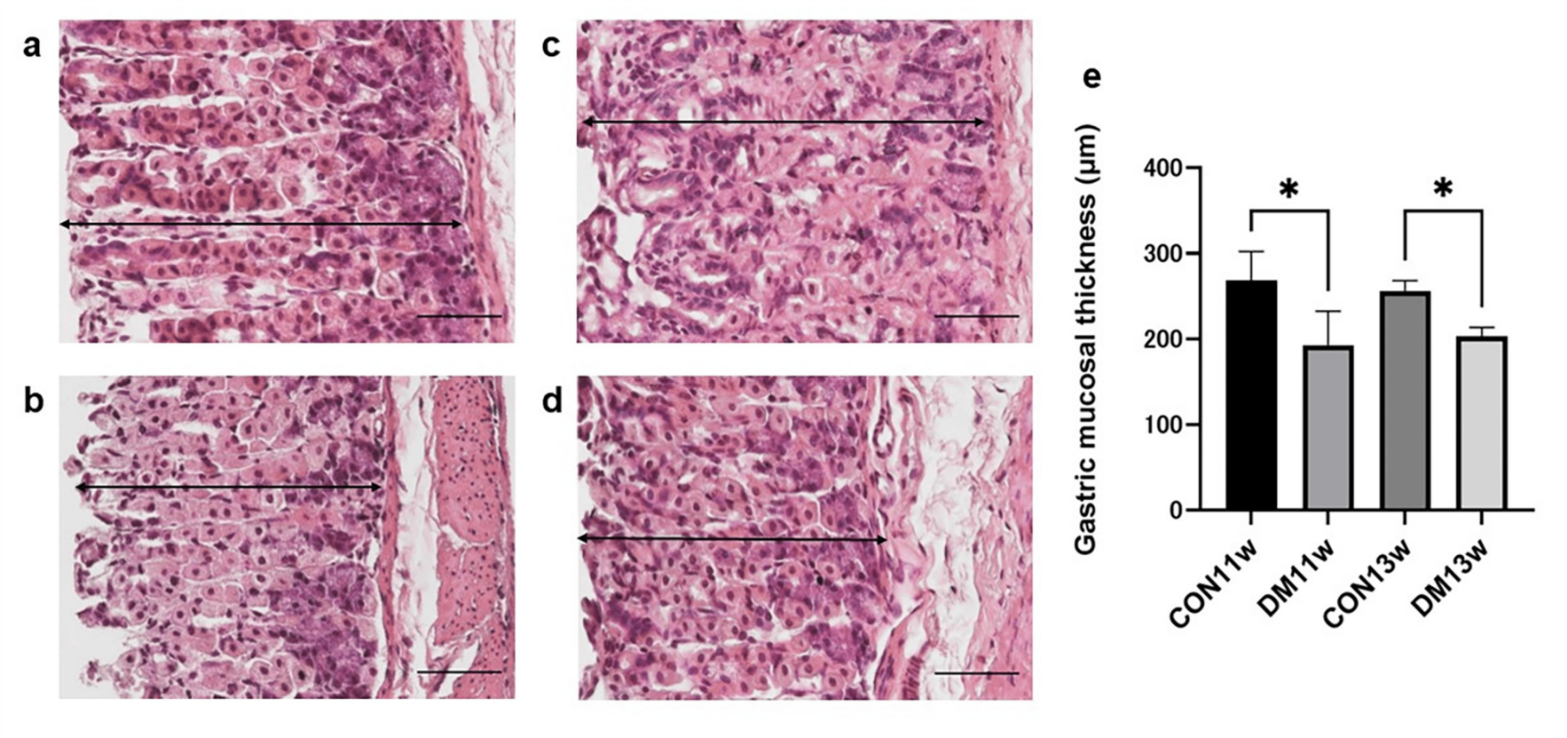
H&E staining images of the stomach in mice from the CON11w (a), DM11w (b), CON13w (c), and DM13w (d) groups, and comparisons of the thickness of the mucous membrane among the four groups (e). Data are shown as mean ± 95% confidence interval (CI) (n = 4 per group at 11 weeks; n = 5 per group at 13 weeks). Two-way ANOVA followed by the Holm–Sidak test was used for multiple comparisons. * p < 0.05. Magnification = 100×; scale bars = 50 μm. CON: control group; DM: type 2 diabetes mellitus group; w: weeks

The thickness of the mucosa was measured by staining the dissected gastric mucosa with H&E to evaluate the tissue structure of the stomach in the DM and CON groups. The mucosa appeared thicker, and the parietal cells were more distinct in the CON groups than in the DM groups (Figures 4a-4d). At 11 weeks, the gastric mucosa was significantly thinner in the DM11w group (mean ± 95% CI: 192.9 (129.6-256.1) μm) than in the CON11w group (268.6 (215.0-322.2) μm; Welch’s t = 2.91, df = 5.84, p = 0.028, Cohen’s d = 2.06).

At 13 weeks, the DM13w group also showed a reduced mucosal thickness (224.3 (206.2-242.4) μm) compared with the CON13w group (256.2 (241.3-271.1) μm; t = 3.78, df = 7.70, p = 0.0058, d = 2.39).

Two-way ANOVA revealed a significant main effect of diabetes (F = 17.39, p = 0.0009, partial η² = 0.55), while age and interaction effects were not significant (Figure 4e).

IHC analysis of ghrelin expression in the stomach

**Figure 5:**
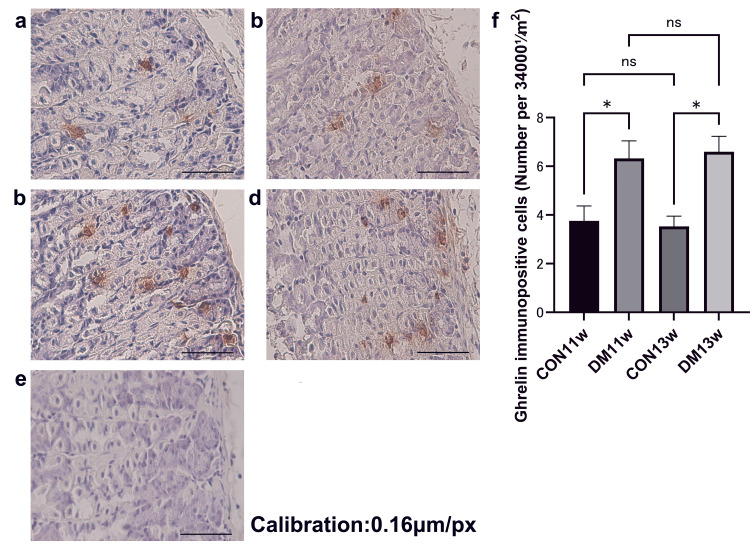
Immunohistochemistry staining of ghrelin in the stomach Representative images from the CON11w (a), DM11w (b), CON13w (c), and DM13w (d) groups. (e) Negative control section processed without primary antibody showing no immunoreactive signal, confirming staining specificity. (f) Quantification of ghrelin-immunopositive cells. Data are shown as mean ± 95% confidence interval (CI) (n = 4 per group at 11 weeks; n = 5 per group at 13 weeks). Two-way ANOVA followed by the Holm–Sidak test was used for multiple comparisons. * p < 0.05. Magnification = 200×; scale bars = 50 μm. ns: not significant; CON: control group; DM: type 2 diabetes mellitus group; w: weeks

IHC staining of the gastric body mucosa revealed ghrelin-positive cells at the base of the gastric fundus at 11 and 13 weeks. In the CON groups, ghrelin-producing cells were observed between the chief and parietal cells in the fundic glands. The number of ghrelin-positive cells was markedly higher in the DM groups than in the corresponding CON groups (Figures 5a-5d). At 11 weeks, the DM11w group showed significantly higher ghrelin-positive cell counts than the CON11w group (mean ± 95% CI: 6.50 (5.21-7.79) vs. 3.85 (2.65-5.05); Welch t = 4.79, df = 5.97, p = 0.0031, Cohen’s d = 3.39).

Similarly, at 13 weeks, the DM13w group had significantly greater counts than the CON13w group (6.23 (5.31-7.15) vs. 3.53 (3.01-4.05); t = 7.09, df = 6.33, p = 0.0003, d = 4.48).

Negative control sections processed without primary antibody exhibited no immunoreactive signals, confirming the specificity of ghrelin staining (Figure 5e).

Two-way ANOVA revealed a significant main effect of diabetes (F = 68.7, p < 0.0001, partial η² = 0.83), whereas age and interaction effects were not significant (Figure 5f).

Gastric ghrelin mRNA expression

**Figure 6:**
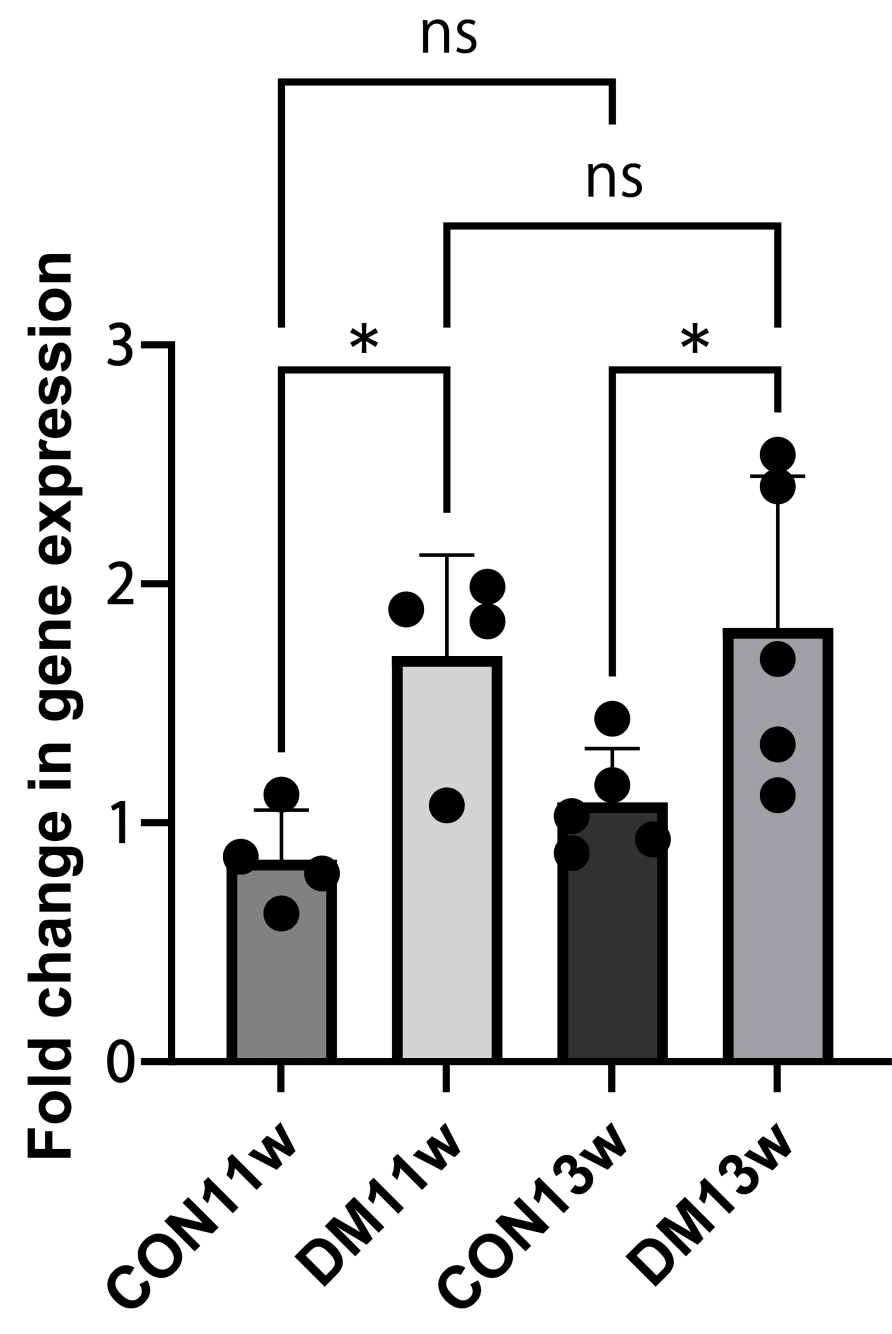
Ghrelin expression level in the stomach of the CON11w, DM11w, CON13w, and DM13w groups Data are shown as mean ± 95% confidence interval (CI). Each dot represents one animal (n = 4 per group at 11 weeks; n = 5 per group at 13 weeks). Two-way ANOVA followed by the Holm–Sidak test was performed (p = 0.013 for DM vs. CON at 13 weeks). * p < 0.05. ns: not significant; CON: control group; DM: type 2 diabetes mellitus group; w: weeks

RT-qPCR using TB Green chemistry was performed to evaluate ghrelin mRNA expression in the stomach. At 11 weeks, ghrelin mRNA expression was slightly lower in the DM11w group than in the CON11w group (mean ± 95% CI: 0.61 (0.43-0.79) vs. 1.00 (0.83-1.17); Welch t = 3.31, df = 5.92, p = 0.013, Cohen’s d = 1.69). At 13 weeks, ghrelin mRNA expression was also significantly lower in the DM13w group than in the CON13w group (0.58 (0.42-0.74) vs. 1.00 (0.84-1.16); t = 3.43, df = 6.01, p = 0.011, d = 1.77). Two-way ANOVA revealed a significant main effect of diabetes (F = 15.8, p = 0.002, partial η² = 0.73) without significant age or interaction effects (Figure 6). These results indicate a robust downregulation of gastric ghrelin gene expression under diabetic conditions, with large effect sizes supporting biological relevance.

Data are presented as mean ± 95% CI, and individual data points are overlaid on group means (Figure 6). Overall, these findings demonstrate a clear downregulation of gastric ghrelin gene expression in diabetic mice, with no significant time-dependent changes within each group.

## Discussion

In this study, we confirmed that ghrelin expression in the gastric mucosa was significantly increased in T2DM mice, representing a major tissue-level alteration in the diabetic stomach. At 13 weeks, the magnitude of the difference was large (RT-qPCR: p = 0.013; Cohen’s d = 1.69, 95% CI (0.10-3.28)), and immunohistochemistry showed large to extremely large effects across time points, indicating biological relevance. T2DM causes various changes in gastric tissue due to prolonged hyperglycemia and metabolic and redox stressors [23]. Studies on mouse models of T2DM have shown atrophy of the gastric mucosa, infiltration of inflammatory cells, and cell apoptosis, suggesting a deterioration of the protective and digestive functions of the stomach [23]. In addition, reduced gastric acid secretion in patients with T2DM may affect hormone secretion and systemic functions [24]. Given these established gastric alterations in diabetes, our tissue-level data reinforce that the diabetic stomach undergoes remodeling concurrently with a robust, localized rise in ghrelin expression. In this study, STZ was administered to nine-week-old mice based on the evidence that STZ sensitivity is reduced in immature and aged mice. Sexually mature mice between eight and 12 weeks of age are considered optimal for T2DM induction [25]. Given that hyperglycemia typically peaks between two and six weeks after STZ administration [26], therefore, analyses at 11 and 13 weeks, corresponding to two and four weeks after administration, provided optimal timing for evaluation. Consistently, hyperglycemia was induced under these conditions, and we confirmed that ghrelin expression in the gastric mucosa was significantly increased, supporting both the validity of the model and the reliability of our findings.

In our study, gastric ghrelin expression was markedly increased, indicating tissue-level adaptation, whereas previous reports have focused on neural and systemic alterations in diabetes. Changes in the enteric, autonomic, and central nervous systems play essential roles in influencing hormone secretion and systemic functions in the pathophysiology of diabetic gastroenteropathy [24]. Hyperglycemia and redox imbalance can damage enteric neurons, inducing apoptosis and alterations in the intestinal microbiota, which can affect hormone secretion and the regulation of systemic functions. In addition, colonic motor dysfunction in human diabetes is associated with enteric neuronal loss and increased oxidative stress [3]. Notably, the impact of T2DM on gastric tissue is relatively under-represented in the literature compared to other organs, such as the liver and kidneys [27], emphasizing the importance of investigating gastric changes and hormone secretion in T2DM. Our findings specifically localize the change to the gastric mucosa, complementing but not replacing prior work on circulating ghrelin or extra-gastric organs. In this study, IHC and RT-qPCR findings confirmed that ghrelin levels in T2DM increased significantly. This is contradictory to previous studies showing decreased circulating ghrelin in T2DM [12, 28], likely due to our focus on gastric tissue analyzed by RT-qPCR. This suggests that tissue-localized ghrelin expression does not necessarily correlate with circulating ghrelin levels. Therefore, we interpret the present increase as compatible with a local adaptive response within the gastric mucosa rather than evidence of a direct causal pathway.

In the present study, we observed a marked increase in gastric ghrelin expression in STZ-induced T2DM mice compared with controls, as shown by both RT-qPCR and immunohistochemistry. T2DM is characterized by decreased plasma ghrelin levels; however, reduced ghrelin and mRNA levels are observed in STZ-induced DM rats [13]. Increased ghrelin sensitivity and signaling may occur due to reduced insulin secretion in STZ-induced T2DM. Elevated plasma ghrelin levels may contribute to diabetic hyperphagia, partially offsetting ghrelin secretion and leading to reduced plasma ghrelin levels. In addition, early-stage STZ-DM shows increased plasma ghrelin levels, which decrease in later stages, potentially related to anorexia, muscle wasting, and delayed gastrointestinal transit in diabetes [29]. Collectively, our results suggest that the increased gastric ghrelin expression observed in our study may reflect a stomach-intrinsic adjustment that can diverge from plasma dynamics, highlighting tissue-specific regulation in diabetes. The reduction in circulating ghrelin levels in T2DM may be associated with the effects of leptin and insulin. However, this was not investigated in the present study. Increased ghrelin signaling in STZ-DM rats, combined with decreased circulating insulin and leptin, activates hypothalamic neuropeptide responses underlying increased food intake in diabetes [29]. Leptin regulates satiety in STZ-DM mice, suggesting that it can reverse elevated plasma ghrelin levels independent of insulin concentration and food intake [30]. Thus, ghrelin and leptin, along with insulin, may be interrelated in diabetes-induced changes in food intake and independent alterations in ghrelin levels. Within this context, our gastric data provide a tissue-level anchor for future integrative studies spanning gastric, endocrine, and neural circuits.

Importantly, we observed decreased gastric mucosal thickness alongside increased ghrelin expression in diabetic mice. This dual alteration suggests that diabetes-related mucosal injury could be consistent with an adaptive rise in local ghrelin expression, without establishing causality. Oxidative stress, which causes cellular damage and inflammation, is known to be exacerbated in patients with T2DM [31]. Ghrelin administration has been suggested to reduce oxidative stress in T2DM rats effectively. Ghrelin exerts anti-inflammatory effects [32] and protects against autophagy [33]. In diabetes, oxidative stress leads to reduced acid secretion in the stomach due to decreased H+-K+-ATPase activity and a decrease in the number of tubules in gastric parietal cells, along with reduced mitochondrial numbers [16]. These pathophysiological changes in diabetes, taken together with our tissue-level findings, motivate testing whether gastric ghrelin metrics serve as biomarkers of diabetic gastric involvement and whether targeted modulation of the gastric ghrelin pathway improves gastrointestinal function.

First, plasma ghrelin (acyl/des-acyl) was not measured, precluding tissue-to-plasma coupling analyses. Second, only male mice were used; potential sex differences remain untested. Third, oxidative stress assays (e.g., MDA, SOD, GSH) and inflammatory panels were not performed, so mechanistic links are hypothesis-generating. Future work should (i) quantify gastric and plasma ghrelin in parallel, (ii) include females to evaluate sex effects, (iii) incorporate oxidative/inflammatory readouts, and (iv) assess the therapeutic modifiability of gastric ghrelin signaling (e.g., via mastication paradigms or pharmacologic tools) with relevance to diabetic gastroenteropathy.

This study demonstrates a significant upregulation of ghrelin mRNA and protein expression within the gastric mucosa of STZ-induced T2DM mice, with large effect sizes indicating biological relevance. By localizing the signal to the stomach, the primary site of ghrelin production, our findings extend a literature largely focused on circulating levels or extra-gastric organs and support evaluation of gastric ghrelin as a biomarker and potential therapeutic target in diabetic gastric dysfunction. At present, we avoid causal claims; instead, we interpret the association as consistent with a local adaptive response that warrants mechanistic and translational testing.

## Conclusions

Immunostaining and RT-qPCR analyses revealed elevated gastric ghrelin levels in T2DM mice. To our knowledge, this is the first report to examine ghrelin-related changes in the gastric mucosa in T2DM. This novel observation underscores the importance of evaluating local gastric expression, which has been largely overlooked compared with circulating levels or other organs such as the pancreas, hypothalamus, and adipose tissue. The consistent upregulation with large effect sizes supports its biological relevance and indicates a tissue-specific adaptive response to diabetic stress. These findings contribute to understanding the changes in gastric mucosa, metabolism, and endocrine function associated with T2DM and suggest a compensatory role of gastric ghrelin in endocrine regulation. They may also provide translational insights that could contribute to the development of novel therapeutic approaches to diabetes.

## Figures and Tables

**Figure 7:**
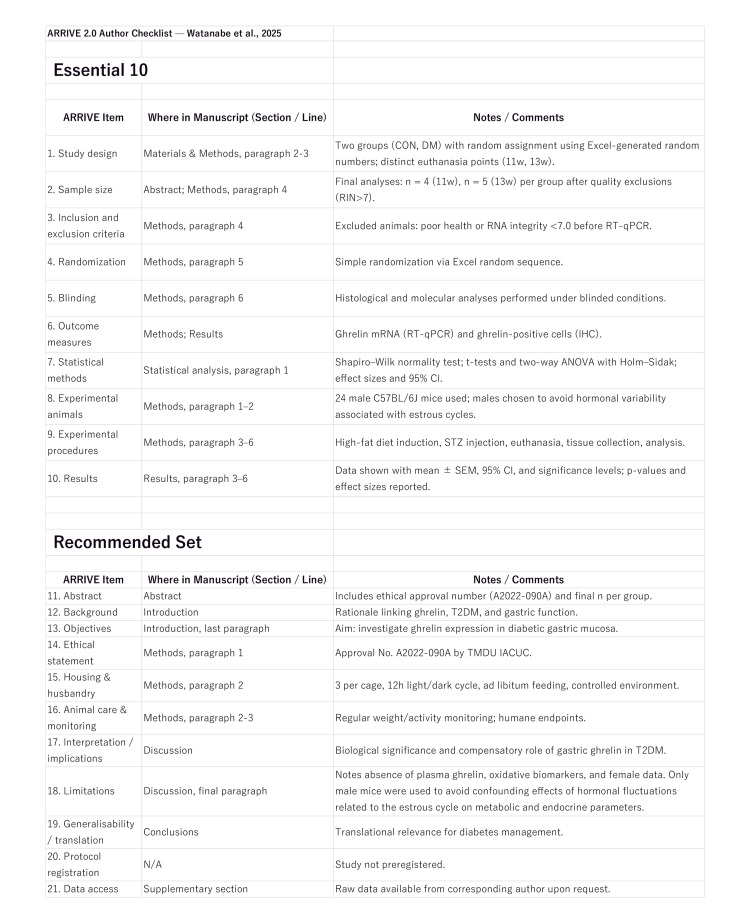
ARRIVE 2.0 Checklist Mapping each item to section/paragraph references in the manuscript. The main text already notes ARRIVE compliance [15]. ARRIVE: Animal Research: Reporting of In Vivo Experiments

**Figure 8:**
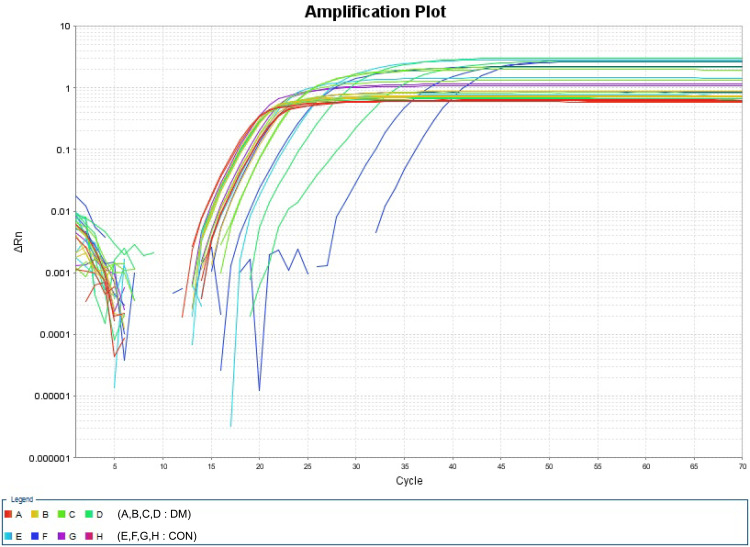
Representative amplification plots for ghrelin and GAPDH genes. Real-time polymerase chain reaction amplification curves generated on the Applied Biosystems 7500 System show consistent exponential amplification and plateau phases across all samples, confirming high amplification efficiency (90–110%) and reproducibility.

## Appendices

Appendix A
